# Cockayne Syndrome-Associated CSA and CSB Mutations Impair Ribosome Biogenesis, Ribosomal Protein Stability, and Global Protein Folding

**DOI:** 10.3390/cells10071616

**Published:** 2021-06-28

**Authors:** Mingyue Qiang, Fatima Khalid, Tamara Phan, Christina Ludwig, Karin Scharffetter-Kochanek, Sebastian Iben

**Affiliations:** 1 Department of Dermatology and Allergic Diseases, Ulm University, Albert-Einstein Allee 23, 89081 Ulm, Germany; mingyue.qiang@uni-ulm.de (M.Q.); fatima.khalid@uni-ulm.de (F.K.); tamara.phan@uni-ulm.de (T.P.); karin.scharffetter-kochanek@uni-ulm.de (K.S.-K.); 2 Bavarian Center for Biomedical Mass Spectrometry, TUM, University of Munich, 85354 Freising, Germany; tina.ludwig@tum.de

**Keywords:** RNA polymerase I, ribosome, Cockayne syndrome, translational fidelity, loss of proteostasis

## Abstract

Cockayne syndrome (CS) is a developmental disorder with symptoms that are typical for the aging body, including subcutaneous fat loss, alopecia, and cataracts. Here, we show that in the cells of CS patients, RNA polymerase I transcription and the processing of the pre-rRNA are disturbed, leading to an accumulation of the 18S-E intermediate. The mature 18S rRNA level is reduced, and isolated ribosomes lack specific ribosomal proteins of the small 40S subunit. Ribosomal proteins are susceptible to unfolding and the CS cell proteome is heat-sensitive, indicating misfolded proteins and an error-prone translation process in CS cells. Pharmaceutical chaperones restored impaired cellular proliferation. Therefore, we provide evidence for severe protein synthesis malfunction, which together with a loss of proteostasis constitutes the underlying pathophysiology in CS.

## 1. Introduction

Cockayne syndrome (CS) is a rare premature aging disease characterized by childhood onset of degenerative symptoms reminiscent of the aging body, including subcutaneous fat loss, alopecia, cataracts, neurological degeneration, and cachexia [1]. These symptoms are accompanied by developmental delay, resulting in a severe phenotype that can lead to childhood mortality. CS is mainly caused by mutations in the genes *ERCC6* and *ERCC8* encoding for CSB and CSA, respectively; additionally, a number of patients with mutations in the *XPB*, *XPD*, *XPG*, and *XPF* genes also display features of CS [2].

CS proteins are involved in the transcription-coupled repair (TCR) branch of the NER pathway explaining the elevated UV sensitivity of the patients and, thus, CS is regarded as a DNA damage disease [3]. However, a homozygous null mutation in CSB was identified in a UV-sensitive (UVs) patient that similarly displayed TCR failure, which is the common hallmark of CS. By contrast, this UVs patient does not display the severe abnormalities associated with CS [4]. Additionally, the total loss of NER is not followed by degenerative features during childhood, suggesting that alternative functions of the CS proteins play a crucial role in the disease pathogenesis [5].

Previously, studies have shown that the CS proteins are involved in transcription by RNA polymerase I in the nucleolus, which is the key step of ribosomal biogenesis [6,7,8]. Ribosomal biogenesis is a complex process that requires the coordinated action of all three RNA polymerases. It starts with the synthesis of the precursor ribosomal RNA (pre-rRNA) by RNA polymerase I, followed by rapid processing of the pre-rRNA and assembly of pre-ribosomal particles, the major biosynthetic process in cells [9].

A recent study by Alupei et al. [10] proposed a novel pathomechanism in CS cells, starting from a disturbed RNA polymerase I transcription activity that affects ribosomal performance and leads to misfolded proteins. The misfolded proteins are oxidized by elevated reactive oxygen species (ROS) and induce an unfolded protein response that represses transcription by RNA polymerase I.

Ribosomes are essential for protein production as well as cellular growth and proliferation. Here, we addressed the question of how the mutations in CS cells affect ribosomal biogenesis in molecular detail. In this study, we demonstrated that RNA polymerase I transcription is affected specifically in CS cells, leading to disturbed pre-rRNA processing. Interestingly, ribosomes isolated from CS cells under stringent conditions display a loss of ribosomal proteins of the small subunit. Moreover, ribosomal proteins from CS, but not from UV-sensitive cells, show an elevated susceptibility to protein unfolding. Of note, rescue experiments with pharmaceutical chaperones (4-phenyl-butyric acid (4PBA) and tauroursodeoxycholic acid (TUDCA)) could restore the retarded proliferation of CS cells.

## 2. Methods

### 2.1. Cell Culture

CS patients’ derived CS1AN SV40-transformed fibroblast [11], CS3BE SV40-transformed fibroblasts [12], and UVsKO SV40-transformed fibroblasts from a UVs patient with a C-to-T homozygous mutation at position 308 of the *ercc6 (CSB)* gene [4] were cultured in Dulbecco’s Modified Eagle Medium (DMEM, Gibco) with 10% fetal bovine serum (FBS, Biochrom), 2 mM l-glutamine, 100 U/mL penicillin, and 100 µg/mL streptomycin (all from Merk Millipore). As controls, CS1AN and CS3BE stably expressing Hämaglutinin (HA)-tagged CSB (HACSB) and CSA (HACSA) proteins, respectively, were used. Additionally, transformed (13O6) human fibroblasts were used as controls. All cells were cultivated under 5% CO_2_ and 3% O_2_ at 37 °C.

### 2.2. Antibodies

Antibodies against RPL13a (2765S) were from Cell Signaling. RPS10 (sc-515655), RPS14 (sc-293478), RPS23 (sc-100837), RPS5 (sc-390935), RPL17 (sc-515904), RPS21 (sc-514411), and HRP-coupled GAPDH (3683) were obtained from Santa Cruz. RPS24 (ab196652) was purchased from Abcam. Rabbit or mouse secondary antibodies coupled to HRP were obtained from Dianova.

### 2.3. RNA Extraction and qRT-PCR

#### 2.3.1. RNA Extraction

Growing CS1AN, CS3BE, UVsKO, HACSB, HACSA, and 1306 fibroblasts cells were harvested by scraping and collected by centrifugation at 2000 rpm for 5 min. The extraction of RNA was performed using an RNeasy Mini Kit (QIAGEN GmbH, Hilden, Germany). The concentration of RNA was measured by using a nanodrop device (Thermo Scientific, Waltham, MA, USA). One µg RNA was reverse transcribed using the Moloney murine leukemia virus Reverse Transcriptase (M170B, Promega, Madison, WI, USA). For primer annealing, 1 µg RNA was pre-incubated with 1 µL random hexamer primer p(dN)_6_ and incubated at 70 °C for 5 min. After annealing, samples were complemented with a reaction mix containing 0.5 µL dNTPs, 0.5 µL RNase inhibitor, 1 µL M-MLV reverse transcriptase, and 5 µL of 5× M-MLV reverse transcription buffer and nuclease-free water up to 25 µL final volume. The reverse transcription was performed for 1 h at 37 °C.

#### 2.3.2. qRT-PCR

Quantitative PCRs were performed using a 7300 Real-Time PCR System (Applied Biosystems, Life Technologies GmbH, Darmstadt, Germany). A total of 100 ng cDNA and FastStart Universal SYBR Green Master (Roche) were used for qRT-PCR. A standard curve of the oligonucleotide of interest with a linear regression with R2 values > 0.8 was used for calculation of the absolute amount (ng) of the oligonucleotide of interest within 100 ng total cDNA. Primers and standard curves used for qPCR analysis are listed in the Supplementary Material.

### 2.4. Ribosome Isolation

Ribosome isolation was performed according to the method of Penzo et al. [13] (Penzo et al., 2016). Briefly, 4 × 10^6^ cells of each cell line were pelleted and lysed by adding 2× packed cell volume of 10 mM Tris-HCl, pH 7.4, 10 mM NaCl, 3 mM MgCl_2_, and 0.5% Nonidet P-40. Following 10 min incubation on ice, the lysates were centrifuged at 16,900× *g* for 10 min at 4 °C. The supernatants were collected, and the ribosomes were pelleted by ultracentrifugation (15 h at 110,000× *g* and 4 °C) through a discontinuous sucrose gradient consisting of 2.25 mL 1.0 M sucrose, and 2.25 mL 0.7 M sucrose, both containing 30 mM Hepes/KOH, pH 7.5, 2 mM magnesium acetate, and 1 mM DTT; KCl was 500 mM in the 0.7 M layer and 70 mM in the 1 M sucrose layer.

### 2.5. Protein Folding and Stability

Protein folding and stability were determined by staining with BisANS dye (4,4′-dianilino-1, 1′-binaphthyl-5, 5′-disulfonic acid, dipotassium salt (Sigma)). Briefly, 5 µg ribosomes were treated with 2 M Urea for 2 h. BisANS (30 µM final concentration) was added in each sample. Fluorescence was measured using a Varioskan^TM^ LUX at an excitation wavelength of 375 nm and 500 nm emission.

### 2.6. Heat Sensitivity Assay

Heat sensitivity analysis was conducted according to the method of Treaster et al. (2014) [14]. In total, 4 × 10^6^ cells were dissolved in a 1.5× packed cell volume of Dignam A buffer and incubated on ice for 15 min. Cells were lysed by passing them 50 times through a 23G syringe and centrifuged for 20 min at 10,400× *g* at 4 °C. The cytoplasmic extracts were subsequently centrifuged by ultracentrifugation for 1 h at 100,000× *g* at 4 °C. In total, 200 μg soluble protein were heat treated at 99 °C for 15 min and immediately centrifuged for 5 min at 16,000× *g*. Pellets were resuspended in 10 μL 4 M urea, and both resuspended pellets and supernatants were quantified using the Bradford assay.

### 2.7. Cell Growth

To evaluate the proliferation kinetics of the drug-treated cell lines, 1 × 10^5^ cells were seeded in six-well dishes with standard media, TUDCA (200 μM) (Millipore) media, or 4PBA (1 mM) (Sigma) media. Cells were counted every 48 h over a period of 16 days using a hemocytometer after they were detached with trypsin. Then, 1 × 10^5^ cells were reseeded, and the growth dynamics were calculated according to the published method by Lin et al [15].

### 2.8. Western Blot

Cells were harvested from 15 cm culture dishes (at 80% density) and lysed with lysis buffer (10% glycine, 1% Triton X 100, 137 Mm NaCl, 20 mM Tris pH 8, 2 mM EDTA pH 8.1). In total, 50 µg lysate and 5 µg ribosomes were separated on 15% SDS-PAGE and transferred to nitro-cellulose blotting membrane (A29434119, GE Healthcare) overnight at 4 °C. Membranes were blocked at room temperature (RT) for 2 h with blocking buffer (5% milk power, 0.1% Tween 20, diluted in phosphate-buffered saline (PBS)), washed with PBS for at least 30 min, and incubated with secondary antibodies at RT for 1 h. The membranes were washed again with PBS before developing on Fusion Fx7 (Vilber). Images were processed and quantified by ImageJ. Using the ImageJ toolbar, we selected the first Western band and plotted the band. When all the peaks have been selected, the area and percent table will appear. For normalization, we repeated the step with the loading control. The data were copied to Excel, and the loading control was used as a denominator to calculate the ratio.

### 2.9. Northern Blot

In total, 5 µg total RNA was denatured at 65 °C for 15 min and immediately placed on ice for 5 min. All samples were separated on a 0.9% agarose gel with 80 V for 3 h. Then, RNAs were transferred to Amersham Hybond membrane overnight. The membranes were crosslinked with 1200 J UV the following day and pre-hybridized with pre-hybridization buffer (50% formamide, 0.1% SDS, 8× Denhards solution, 5× SSC buffer, 50 mM NaP buffer, 0.5 mg/mL t-RNA) for 2 h at 65°. The 32P-labeled oligonucleotide probe was denatured at 95 °C for 10 min and added to the membrane. Following one hour at 65 °C, the membrane was subsequently incubated at 37 °C overnight. Following washing in 2xSSC, the membrane was exposed to an X-ray film and quantified after Wang and Pestov [16] using ImageQuant Software. In brief, we determined the total volume of the hybridization signal in the bands corresponding to pre-rRNA species detected with each probe. 47Spre-rRNA was used as a convenient denominator in calculating precursor ratios and GraphPad Prism to show the standard deviation of each ratios. In Excel, the values obtained from the same lane and in the same hybridization were used to calculate the ratios. The ratios were transformed (log2) and then subtracted from the corresponding values. The log average and standard deviation for the biological replicas was calculated. The normalized log average values were plotted according to a predetermined order along the Y-axis. The used probes were ITS1 (5′GGGCCTCGCCCTCCGGGCTCCGTTAATGATC3′) and ITS2 region (5′CTGCGAGGAACCCCCAGCCGCGCA3′).

### 2.10. Mass Spectrometry

Quantitative proteomics. Ribosomes from CS^mut^, CS^rec^, and UVsKO cells were analyzed using quantitative proteomics. Per sample, ca. 5 µg of isolated ribosomes were resuspended in 50 mM Tris HCl pH 8.0, 2 mM magnesium acetate, and 100 mM ammonium acetate. The samples were reduced, alkylated, and digested with trypsin (Promega). The generated peptides were desalted and measured on a Dionex Ultimate 3000 RSLCnano system coupled to a Q-Exactive HF-X mass spectrometer (Thermo Fisher Scientific). A 50-minute linear gradient from 4% to 32% of solvent B (solvent A: 0.1% formic acid in water and 5% (*v*/*v*) DMSO; solvent B: 0.1% formic acid in acetonitrile and 5% (*v*/*v*) DMSO) was applied. The Q-Exactive HF-X mass spectrometer was operated in data-dependent acquisition (DDA) and positive ionization mode. MS1 spectra (360–1300 m/z) were recorded at a resolution of 60,000 using an automatic gain control (AGC) target value of 3e6 and maximum injection time (maxIT) of 45 ms. Up to 18 peptide precursors with charge state 2 to 6 were selected for fragmentation and dynamic exclusion of 25 s was enabled. Peptide fragmentation was performed using higher energy collision-induced dissociation (HCD) and a normalized collision energy (NCE) of 26%. The precursor isolation window width was set to 1.3 m/z. MS2 resolution was 15,000 with an automatic gain control (AGC) target value of 1e5 and a maximum injection time (maxIT) of 25 msec.

Peptide identification and quantification were performed using the software MaxQuant (version 1.6.3.4) with its built-in search engine Andromeda [17,18]. MS2 spectra were searched against a Uniprot-derived human reference proteome (UP000005640, 20,353 reviewed entries, downloaded 17.7.2020) supplemented with common contaminants (built-in option in MaxQuant). Trypsin/P was specified as proteolytic enzyme. Precursor tolerance was set to 4.5 ppm, and fragment ion tolerance to 20 ppm. Results were adjusted to 1% false discovery rate (FDR) on peptide spectrum match (PSM) level and protein level employing a target-decoy approach. The minimal peptide length was defined as seven amino acids, and the “match-between-run” function was disabled. Carbamidomethylated cysteine was set as fixed modification and oxidation of methionine and N-terminal protein acetylation were set as variable modifications. To compare the relative protein abundances between the ribosome samples, label-free quantification (LFQ) values were used. 

Mass spectrometry analyses of isolated ribosome from the CS3BE and CS1AN cell lines and respectively the HACSA and HACSB reconstituted cell lines were performed by the Bavarian Center for Biomolecular Mass Spectrometry, Technical University Munich. The quantification of the mass spectrometry data was performed using Excel and program software Rstudio.

### 2.11. Statistical Analysis

Statistical analysis was performed using GraphPad Prism (GraphPad 6 software). Each experiment was performed independently at least three times. Data are shown as mean ± standard deviation (SD). Statistical significance was calculated using an unpaired two-tailed Student’s *t*-test in GraphPad Prism software. Asteriks (*) in the figures represent ρ values (* *p* < 0.05, ** *p* < 0.01, *** *p* < 0.001).

## 3. Results

### 3.1. RNA Polymerase I Transcription and Processing Disturbances in CS Cells

**Figure 1:**
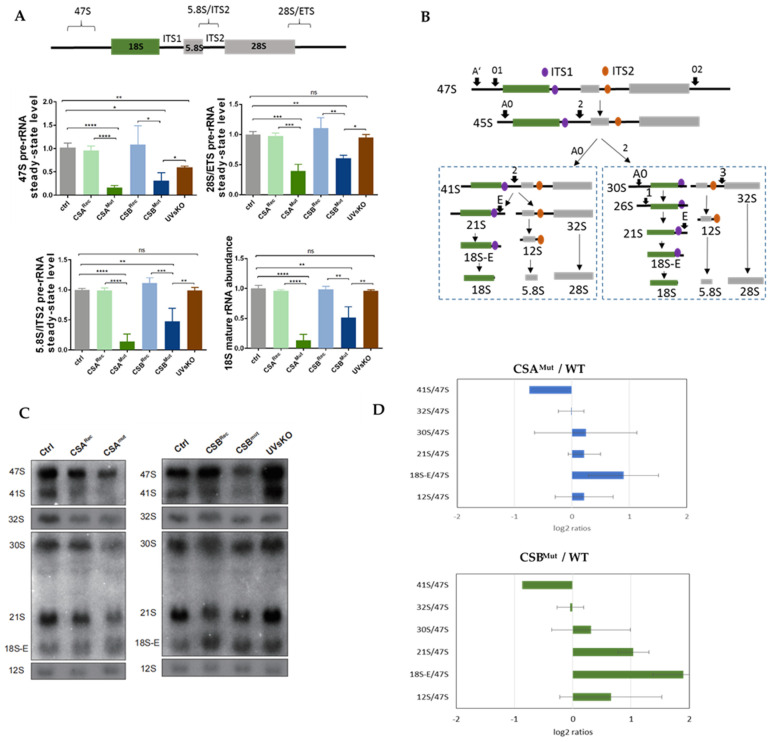
Deficient RNA Pol I transcription and altered rRNA processing in CS cells. (**A**) Quantitative PCR analysis of 47S, 28S/ETS, 5.8S/ITS2, and mature 18S rRNA. Used cell lines were wt (1306 fibroblasts), CSA^rec^ (CS3BE transfected with HA-CSA), CSA^mut^ (CS3BE), CSB^rec^ (CS1AN, transfected with HA-CSB), CSB^mut^ (CS1AN), and UVsKO (UV-sensitive cells). A scheme of the primer positions is provided above. (**B**) Schema of the rRNA processing pathway in human cells, adapted from Mullineux and Lafontaine [25]. The ITS1 probe is identified in purple; the ITS2 probe is in orange. (**C**) Northern blot image of control cells, CS mutated cells, and reconstituted cells. (**D**) RAMP analysis of the Northern blots reveals a relative accumulation of processing intermediates in CS patient cells. Quantification and statistics are provided in the Supplementary Material (Figure S1). Asteriks (*) in the figures represent ρ values (* *p* < 0.05, ** *p* < 0.01, *** *p* < 0.001, **** *p* < 0.0001).

To investigate ribosomal biogenesis in CS, we used immortalized fibroblasts from CSA (CS3BE) and CSB (CS1AN) CS patients expressing truncated CS proteins [11,19] and compared them with the respective wild-type (wt) gene reconstituted cells (CSA^rec^, CSB ^rec^). As additional controls, we used an immortalized wt cell line and the immortalized CSB null mutant UVsKO cell line from a patient that displayed a DNA repair defect with skin photosensitivity but without the developmental or premature aging features of CS [4]. In a recent publication, Alupei et al. [10] described a significant reduction of 47S pre-rRNA precursor synthesis in CS cells. The primers used to amplify the unprocessed 47S precursor bind the 5′external transcribed spacer that is cleaved in the first processing step A’. Therefore, the 47S qPCR relative values reflect the initiation rate of RNA polymerase I transcription activity and showed a clear reduction in CS cells (Figure 1A). Initiation and elongation are differentially regulated in RNA polymerase I transcription [20]. Monitoring transcription by the amplification of gene-internal regions of the pre-rRNA (5.8S/ITS2, 28S/ETS) and pre-rRNA processing intermediates revealed that not only transcription initiation is impaired in CS, but also transcription elongation or processing dynamics are affected by mutations in CSA and CSB, confirming previous reports [21,22]. The severely reduced RNA polymerase I transcription activity in CS cells translates to a reduced abundance of the mature 18S rRNA, as presented in Figure 1A, but not of the mature 28S rRNA (Supplementary Materials). Interestingly, the null mutation in CSB (UVsKO) reduced the initiation of RNA polymerase I transcription but displayed no influence on the RNA polymerase I gene-internal transcription activity and was not followed by a reduction of the mature 18S rRNA. This result confirms that the RNA polymerase I gene-internal transcription activity impacts on the 18S maturation pathway of the pre-rRNA precursor [23]. Subsequently, Northern blotting was applied to assess the processing dynamics of the pre-rRNA precursor that is cleaved and matured in a multistep process, as schematically depicted in Figure 1B. The corresponding membranes were incubated with a radiolabeled probe (internal transcribed spacer 1, ITS1 and ITS2), and the resulting autoradiographic images (Figure 1C) were densitometrically quantified. The obtained values were normalized to 47S pre-rRNA expression and indicate either if the co-transcriptional processing follows control dynamics or if processing intermediates are underrepresented or accumulate. As presented in the RAMP analysis in Figure 1D and quantification in the Supplemental Figure S1B, mutation in CSA reduced the 41S/47S ratio and increased the 18S-E/47S ratio, suggesting a disturbed processing in CSA mutant cells. Accordingly, truncating mutation of CSB in CS1AN cells but not the complete loss of CSB in UVsKO exhibited a severe impact on pre-rRNA processing (Figure 1D). These results indicate that the RNA polymerase I elongation inhibition by the truncated CSB [21] (Figure 1A) translates to pre-rRNA processing defects as already described for the related disease trichothiodystrophy [24]. Taken together, the truncating mutations in CSB and CSA disturb transcription activity by RNA polymerase I and processing of the pre-rRNA.

### 3.2. Isolated Ribosomes from CS Cells Display Stability Defects

**Figure 2:**
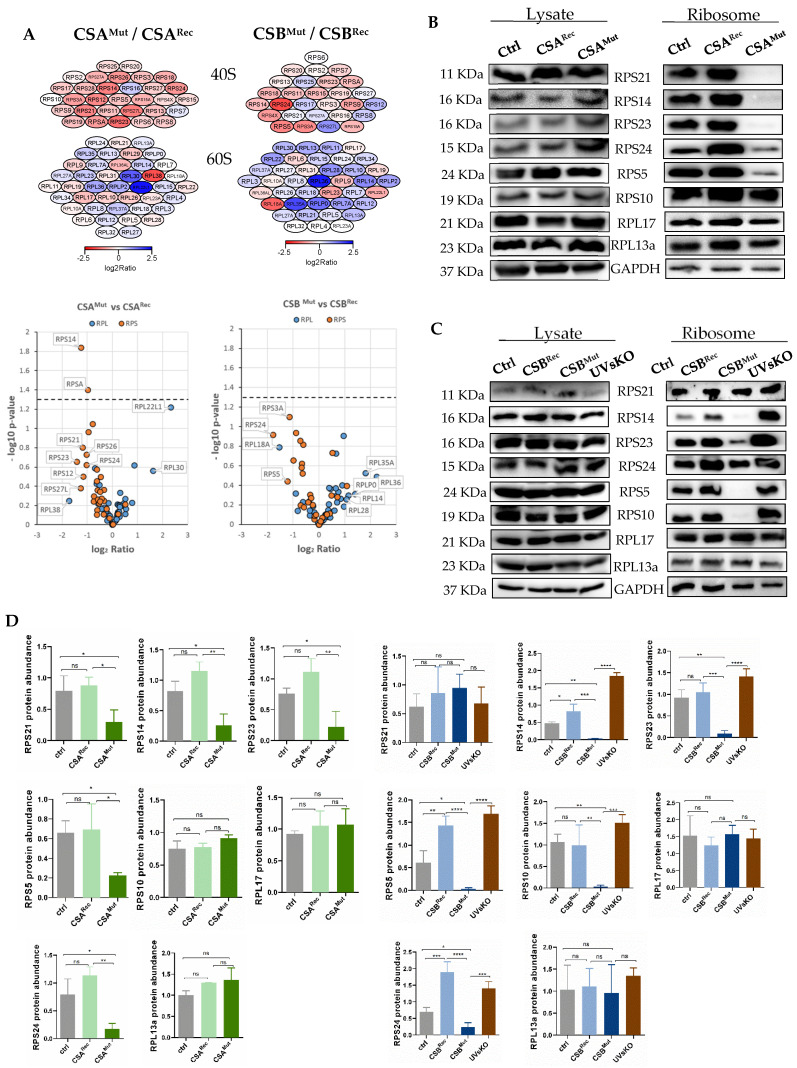
Reduced stability of ribosomes in CS cells. (**A**) Mass spectrometric analysis shows protein intensity of isolated ribosomes of CS-deficient cells compared to reconstituted cells (above), quantification by volcano plot of the small 40S ribosome subunit (red), and the large 60S ribosome subunit (blue) (below). Additional MS analysis is provided in the Supplementary Material (Figure S2). (**B**) Western blot analysis of eight selected ribosomal proteins of whole cell lysates (left) and isolated ribosomes (right) of CSA mutated cells, CSA reconstituted cells, and control cells. GAPDH was used as a loading control. (**C**) Western blot analysis of selected ribosomal proteins of whole cell lysates (left) and isolated ribosomes (right) of CSB mutated cells, CSB reconstituted cells, and control cells. RPL17 and GAPDH were used as loading controls. (**D**) Quantification of Western blots of selected ribosomal proteins in CSA and control ribosomes (left) and (E) CSB and control ribosomal preparations (right). Quantification of ribosomal protein abundance in lysates is provided in the Supplementary Material (Figure S3). Asteriks (*) in the figures represent ρ values (ns, *p* > 0.05, * *p* < 0.05, ** *p* < 0.01, *** *p* < 0.001, **** *p* < 0.0001).

Processing and assembly of pre-ribosomal particles occur co-transcriptionally in a coordinated manner [26]. Hypothesizing that a disturbed transcription and processing of the pre-rRNA precursor translates to the stability of the synthesized ribosomes, we purified ribosomes under stringent conditions [13]. Cytoplasmic extracts isolated from the same number of cells were subjected to sucrose-cushion centrifugation in a high-salt buffer (500 mM KCl). Subsequently, ribosomes were washed, suspended in buffer, and the concentration was determined from the OD260. The same amount of ribosomes was thereafter subjected to an initial mass spectrometry screen, and the relative abundance of the ribosomal proteins was compared between CS and reconstituted cells. Several proteins of both the small and the large ribosomal subunits were underrepresented in the ribosomes of patients’ cells (CSA^MKut^/CSB^Mut^), as shown in the heatmaps (dark red color) and volcano plots of Figure 2A. These data suggest that impaired processing of the rRNA is translated into assembly/stability defects, resulting in ribosomal subunits with non-stoichiometric ribosomal proteins. Alternatively, it may suggest that mutations in CSA or CSB profoundly impact on the gene expression rate of the ribosomal protein subunits. To distinguish between these possibilities, the abundance of underrepresented ribosomal proteins was compared in lysates and in purified ribosomes by Western blotting. The protein content was normalized to RPL13 and RPL17, which are ribosomal proteins that did not display differences in the mass spectrometric analyses and additionally to GAPDH, which was co-purified with ribosomes (Figure 2B). Interestingly, several proteins of the small subunit 3 were almost non-detectable in ribosomal preparations of the CSA mutant cell line (right panels), thus confirming the results of mass spectrometry, whereas they were abundant in the whole-cell lysate (left panels). Western blotting of ribosomal preparations of CSB mutant CS1AN cells revealed a significantly reduced content of several proteins of the 40S small ribosomal subunit). These data imply that a disturbed ribosomal biogenesis results in the dysregulation of ribosomal assembly or stability.

### 3.3. Reduced Heat and Unfolding Stability in CS cells

**Figure 3:**
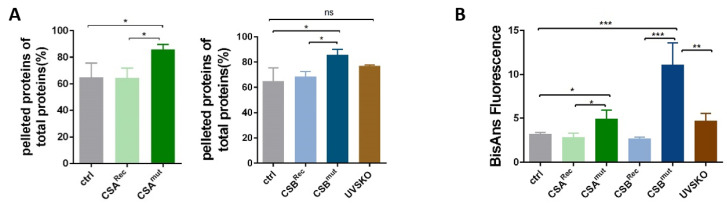
(**A**) Heat sensitivity analysis of cytoplasmic extract from both CS mutated cells and reconstituted cells and from control cells. (**B**) Ribosomal protein unfolding stability was determined by staining with BisANS dye after treatment with 2 M urea. Asteriks (*) in the figures represent ρ values (ns, *p* > 0.05, * *p* < 0.05, ** *p* < 0.01, *** *p* < 0.001).

Previously, employing the identical cell lines as used here, Alupei et al. showed that the fidelity of the translation process is reduced in CS cells [10]. CS ribosomes produce misfolded proteins that are prone to oxidation. In an attempt to further characterize the proteome of CS cells, we investigated the stability of the proteome against heat denaturation. Cytoplasmic extracts were centrifuged to remove insoluble proteins, and the supernatant was subjected to a short heat treatment of 99 °C for 15 min. Subsequent centrifugation separated the heat-denatured fraction from the heat-stable fractionthat was quantified by Bradford measurement. As presented in Figure 3A, the CSA and CSB mutations were associated with a nearly 20% increase in heat-denaturable proteins, supporting and extending previous data [10]. Moreover, this increase in the heat sensitivity of the proteome may imply that errors in the translation process in CS also affect a higher proportion of total cytoplasmic proteins that are misfolded and prone to heat stress. To investigate whether this increase in misfolded proteins also affects the ribosome, we analyzed ribosomes for their unfolding stability. Unfolding stability against heat and urea characterizes long-living species [14]. When challenging ribosomal proteins with urea with subsequent quantification of the amount of unfolding by BisAns incorporation (Figure 3B), we found a profound increase in exposed and labeled hydrophobic side chains. These data highlight that ribosomal proteins of CS cells are highly susceptible to urea-induced unfolding, which is indicative of misfolded proteins in the isolated ribosomes of CS cells. Therefore, the proteome and the ribosomal proteins themselves are characterized by the accumulation of instable variants.

### 3.4. Pharmaceutical Chaperones Enhance Cellular Proliferation

**Figure 4:**
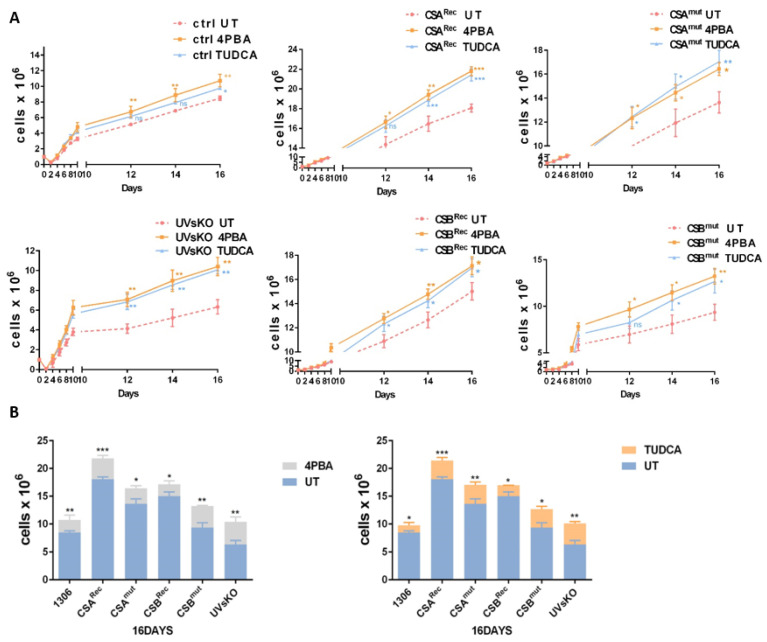
Chemical chaperones increase cell growth. (**A**) Proliferation kinetics of all cell lines in standard media (untreated, UT), 4PBA (1 mM) media, or TUDCA (200 μM) media. Cells were counted every second day and continued over a time period of 16 days. (**B**) Cell number of all cell lines cultured in normal media compared to that in 4PBA media (left) and in TUDCA media (right) after 16 days. Asteriks (*) in the figures represent ρ values (ns, *p* > 0.05, * *p* < 0.05, ** *p* < 0.01, *** *p* < 0.001).

Pharmaceutical chaperones such as 4PBA and TUDCA display the capacity to reduce endoplasmic reticulum (ER) stress and normalize the repressed ribosomal biogenesis and protein synthesis in CS cells [10]. Interestingly, these chaperones were shown to block the hypersensitivity of CS cells to oxidative stress, indicating that protein oxidation might be the driver of apoptosis after oxidative challenge in CS [10]. To investigate whether pharmaceutical chaperones may also restore cellular growth, we performed proliferation kinetics in the presence and absence of 4PBA or TUDCA. Treatment of CS patients’ cells enhanced proliferation to the level of the untreated reconstituted control cells (Figure 4A). Remarkably, control cells and the cells from the mildly affected UVsKO patient also revealed enhanced proliferation in the presence of 4PBA or TUDCA. This might indicate that protein misfolding to a minor extent also occurs in cell culture and thus profits from chaperone treatment. This chaperone experiment further underscores that cellular proliferation is not exclusively responsible for clinical features of growth retardation. This conclusion is based on the observation that in stark contrast to CS patients, UVsKO patients do not suffer from growth retardation [4]. The low toxicity of the used chaperones would make them promising candidates for clinical trials.

## 4. Discussion

CS is a developmental and degenerative disorder with symptoms of the aging body, including neurodegeneration, cataract formation, subcutaneous fat loss, and cachexia. The underlying pathophysiology remains a matter of intense debate because the causative mutations affect genes that code for multifunctional proteins [5]. Other investigators as well as ours have shown that a shared function of the CS proteins is that they are all involved in ribosomal biogenesis by RNA polymerase I [6,7,8,24,27,28]. Here, we propose the hypothesis that disturbances of RNA polymerase I transcription activity impact on pre-rRNA processing and ribosomal assembly. The present study was conducted with immortalized fibroblasts from CSA and CSB mutant CS patients and as controls, the reconstituted cells, a wt cell line, and a cell line from a UV-sensitive patient with normal development and without childhood degeneration. This final cell line displays a DNA-repair defect indistinguishable from CS cells and a null mutation in CSB [4]. By contrast, the CSB CS line used expresses a truncated CSB that represses elongation by RNA polymerase I [21]. CSA and CSB have been shown to influence RNA polymerase I transcription elongation and gene occupancy by nucleolin regulation [22]. Nonetheless, a complete lack of CSB in the UVsKO cell line reduces the initiation of transcription by RNA polymerase I, as it has also been demonstrated for the knockdown of CSB [22] but has no impact on the later steps of RNA polymerase I transcription and mature 18S abundance. These data might imply that elongation by RNA polymerase I transcription and maturation of the primary transcript might impact on the severity of CS disease, but that requires further elongation analysis. Processing of the pre-rRNA precursor is co-transcriptionally regulated, and the dynamics of transcription elongation influence the subsequent cleavage [23]. Indeed, Northern blotting and quantification of the processing intermediates clearly revealed a disturbed processing in CS cells but not in the UVsKO cell line. Ribosomal biogenesis is a highly complex process involving rRNA modifications and the action of at least 200 ribosomal biogenesis factors (RBFs). Moreover, a screen to identify factors required for the maturation of the small 40S subunit revealed that additional to the RBFs, proteins involved in transcription, pre-mRNA splicing, translation, and protein degradation are necessary for pre-40S maturation [29]. This indicates a crosstalk between gene expression/proteome stability pathways and ribosomal biogenesis and is in line with our observation that DNA repair/transcription factors such as CSA and CSB influence ribosomal maturation pathways. 

The assembly of the ribosomal proteins with the rRNA also occurs co-transcriptionally [26] and might be affected by transcriptional disturbances. By mass spectrometric analysis and subsequent Western blotting, we assessed the abundance of ribosomal proteins in isolated ribosomes. Proteins of the small, decoding ribosomal subunit were underrepresented in ribosomal preparations, although they were present at control levels in cell lysates. This finding indicates either disturbed ribosomal assembly or a reduced stability of ribosomes. Ribosomal proteins do not directly participate in peptide bond formation during protein synthesis, but they play essential roles in ensuring correct rRNA folding to enable catalysis [30]. To unravel possible position-specific effects of the underrepresented ribosomal proteins in the structure of the small 40S subunit, we analyzed the relative position of the underrepresented proteins in a 3D structural model (rcsb/4UG0/1). There was no specific region of the 40S subunit identifiable nor an interaction of underrepresented ribosomal proteins, suggesting that it might not be a specific maturation step that is affected by CS mutations. However, we are still ignorant about the exact nature of the defect leading to the loss of ribosomal proteins.

The ribosomal preparations from CS patient cells display less stress resistance to unfolding by urea (Figure 3B) than preparations from control cells. This could imply that the ribosomal proteins might already be misfolded when assembled to unstable ribosomes. Mutations in ribosomal proteins are most common in Diamond–Blackfan anemia (DBA), which is a syndrome of bone-marrow failure and elevated cancer incidence that apparently does not appear to resemble CS. However, the major difference between DBA and CS is the alteration or lack of the ribosomal proteins that might have extraribosomal functions suppressing cancer development [31]. Moreover, it has been shown that mutations in ribosomal proteins can lead to a reduced translational fidelity in DBA [32] but also in other developmental syndromes that resemble CS [33]. Translational fidelity is severely affected in CS, as recently shown in transfection assays by Alupei et al., and it might explain the observed loss of proteostasis with an increase of heat-sensitive proteins and the detection of misfolded proteins in the ribosomes themselves. Whether misfolded ribosomal proteins contribute to the reduced translational fidelity awaits further analysis.

Ribosomopathies causing neurodevelopmental disease in childhood is an emerging concept. They are due to mutations in ribosomal proteins, disturbed factors regulating ribosomal biogenesis involving pre-rRNA processing, and transcription factors of RNA polymerase I (UBTF) or in the enzyme itself (PolR1A/RPA194) [34]. These syndromes are characterized by neurodegeneration with dysmorphic features, developmental retardation, and short stature, which are features that are also characteristics of CS.

The pharmaceutical chaperones shown to stimulate cellular growth (4PBA and TUDCA) were also used in the former study by Alupei et al. and reliably restored impaired transcription initiation by RNA polymerase I transcription and protein synthesis. Remarkably, the pharmaceutical chaperones have the capacity to prevent oxidative stress induced by CS cells apoptosis, likely implying that not unrepaired oxidative DNA damage [35] but rather misfolded and oxidized proteins trigger apoptosis in CS [10]. Since the oxidative hypersensitivity distinguishes CS cells from UVs cells, this hypersensitivity distinguishes severe developmental diseases from mild skin disease. The elevated ROS released by dysfunctional mitochondria in CS [36,37] could be a consequence of unrepaired DNA damage. However, the UVsKO line showed the highest ROS release [10] and might thus also be affected by oxidative DNA damage and mitochondrial dysfunction. However, UVsKO cells display neither ribosomal instability nor a loss of proteostasis; thus, it is tempting to speculate that these cellular pathologies impact on childhood degeneration and premature aging in CS. In summary, our data revealed profound disturbances in ribosomal biogenesis as highlighted in the table below, and the application of chaperones holds strong promise for the treatment or prevention of clinical symptoms.

## Data Availability

Proteomics raw data, MaxQuant search results, and the used protein sequence database have been deposited with the ProteomeXchange Consortium via the PRIDE partner repository (https://www.ebi.ac.uk/pride/) and can be accessed using the data set identifier (reviewer account username: <reviewer_pxd024478@ebi.ac.uk, password: kMUcNSVn).

## Supplementary Materials

The following are available online at https://www.mdpi.com/article/10.3390/cells10071616/s1, Figure S1: Delayed rRNA processing and disturbed ribosomal composition in CS cells, Figure S2: (A) Mass spectrometric analysis shows protein intensity of isolated ribosomes of CSB-deficient cells compared to UVsKO cells, and quantification by volcano plot of the small 40S ribosome subunit and the large 60S ribosome subunit. (B) A bar plot of MS data with p-values. (C) List of unique peptides/RP for label-free MS quantification; Figure S3: Quantification of Western Blots of ribosomal proteins in lysates.
