# Supplementary material for: Cockayne Syndrome-Associated CSA and CSB Mutations Impair Ribosome Biogenesis, Ribosomal Protein Stability, and Global Protein Folding
Source: Cells. 2021 Jun 28;10(7):1616. doi: 10.3390/cells10071616 (PMC8306422; doi:10.3390/cells10071616)
Supplement: Supplementary file 1 [file cells-10-01616-s001.zip › cells-1232823-supplementary.pdf]

Supplemental Figures

Figure S1

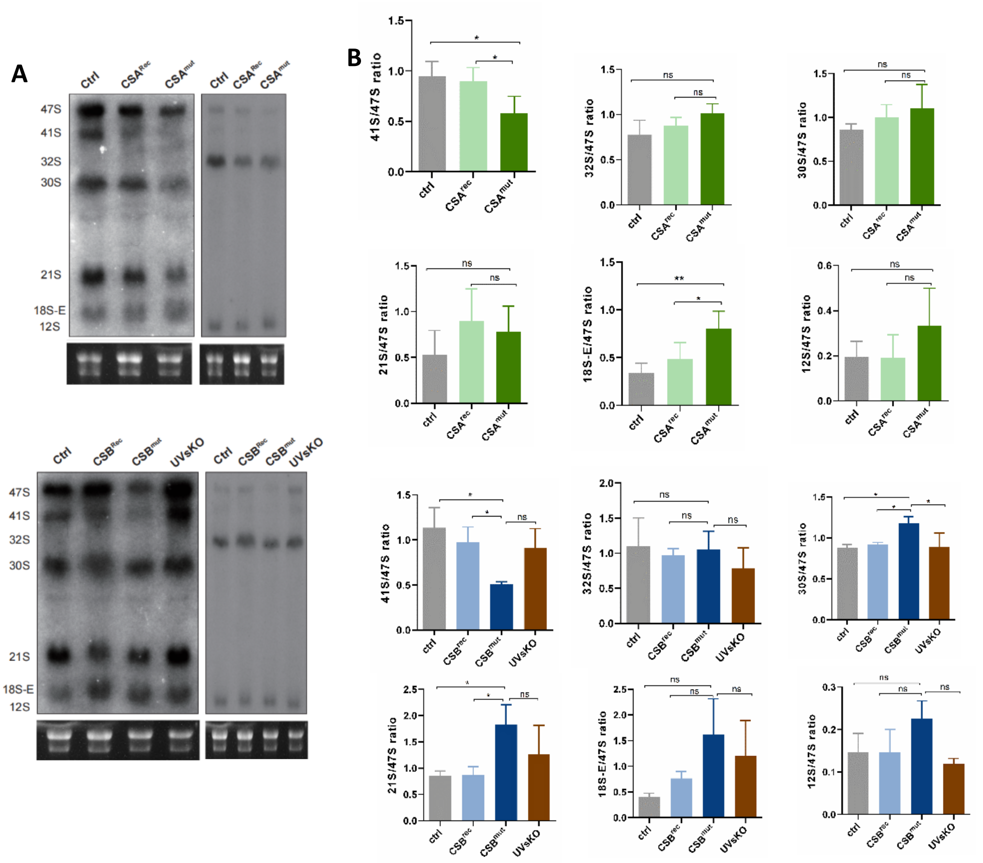

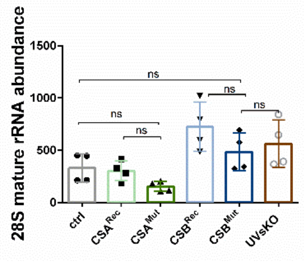

D **qPCR program setting**

Step	Temperature	Time
Denaturation	95 °C	10 seconds
Annealing	60 °C -68 °C	30 seconds
Elongation	72 °C	30 seconds

E

Primer	Forward	Reverse
47S	*TGTCAGGCGTTCTCGTCTC*	*AGCACGACGTCACCACATC*
18S	*CGATGCGGCGGCGTTATTCC*	*GAACGGCCATGCACCACCAC*
28S	*AGTCGGGTTGCTTGGGAATGC*	*CCCTTACGGTACTTGTTGACT*
28S/ETS	*ACCTGGCGCTAAACCATTCGT*	*GGACAAACCCTTGTGTCGAGG*
5.8S/ITS2	*TCGTGCGTCGACGCAG*	*ATTGATCGGCAAGCGACGCTCAG*

Figure S1. (A) Full image of a northern blot analysis in all cell lines. Membranes were hybridized with ITS1 probe, further stripped and re-probed with ITS2. (B) Quantification of different intermediates of pre-rRNA species normalized to the 47S level. (C) Relative quantification of the mature 28S rRNA abundance in CS cells. Values are represented as mean ± SD of at least three independent experiments (*=p<0.05, **=p<0.01, ***=p<0.001). (D) QPCR was run with the different annealing temperatures **60°C (28S/ETS)**, **64°C (5.8/ITS2, 18S)** and **68°C (47S)**. (E) Primer list used in this study

Figure S2

A

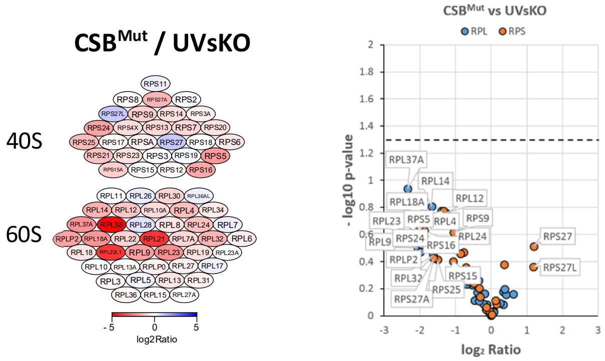

B

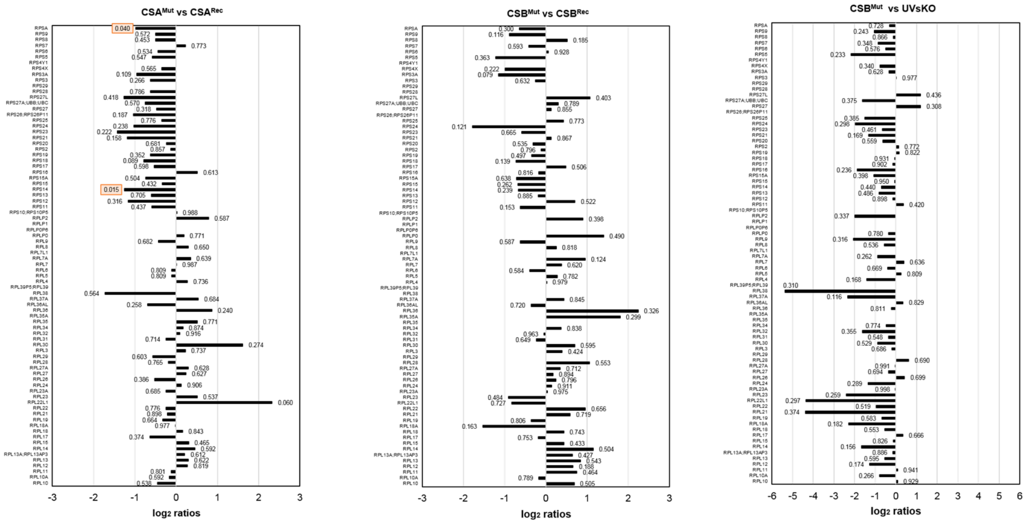

C

Protein names RPL	Peptide counts (unique)
RPL10	12
RPL10A	13
RPL11	10
RPL12	7
RPL13	9
RPL13A;RPL13AP3	10;5
RPL14	5
RPL15	11
RPL17	8
RPL18	10
RPL18A	6
RPL19	6
RPL21	7
RPL22	6
RPL22L1	3
RPL23	8
RPL23A	12
RPL24	7
RPL26	2
RPL27	8
RPL27A	6
RPL28	9
RPL29	2
RPL3	21
RPL30	8
RPL31	8
RPL32	8
RPL34	6
RPL35	5
RPL35A	2
RPL36	6
RPL36AL	2
RPL37A	4
RPL38	6
RPL39P5;RPL39	1;1
RPL4	22
RPL5	15
RPL6	18
RPL7	20
RPL7A	21
RPL7L1	4
RPL8	11
RPL9	13
RPLP0	7
RPLP0P6	1
RPLP1	3
RPLP2	11

Protein names RPS	Peptide counts (unique)
RPS10;RPS10P5	5;3
RPS11	13
RPS12	9
RPS13	8
RPS14	11
RPS15	4
RPS15A	7
RPS16	10
RPS17	9
RPS18	10
RPS19	8
RPS2	15
RPS20	4
RPS21	6
RPS23	7
RPS24	5
RPS25	6
RPS26;RPS26P11	5;4
RPS27	3
RPS27A;UBB;UBC	4;0;0
RPS27L	2
RPS28	2
RPS29	3
RPS3	21
RPS3A	20
RPS4X	7
RPS4Y1	2
RPS5	14
RPS6	11
RPS7	12
RPS8	12
RPS9	12
RPSA	15

Figure S2. (A) Mass spectrometric analysis shows protein intensity of isolated ribosomes of CSB-deficient cells compared to UVsKO cells, and quantification by volcano plot of the small 40S ribosome subunit and the large 60S ribosome subunit. (B) A bar plot of MS data with p-values (C) List of unique peptides/RP for label-free MS quantification.

Figure S3

A

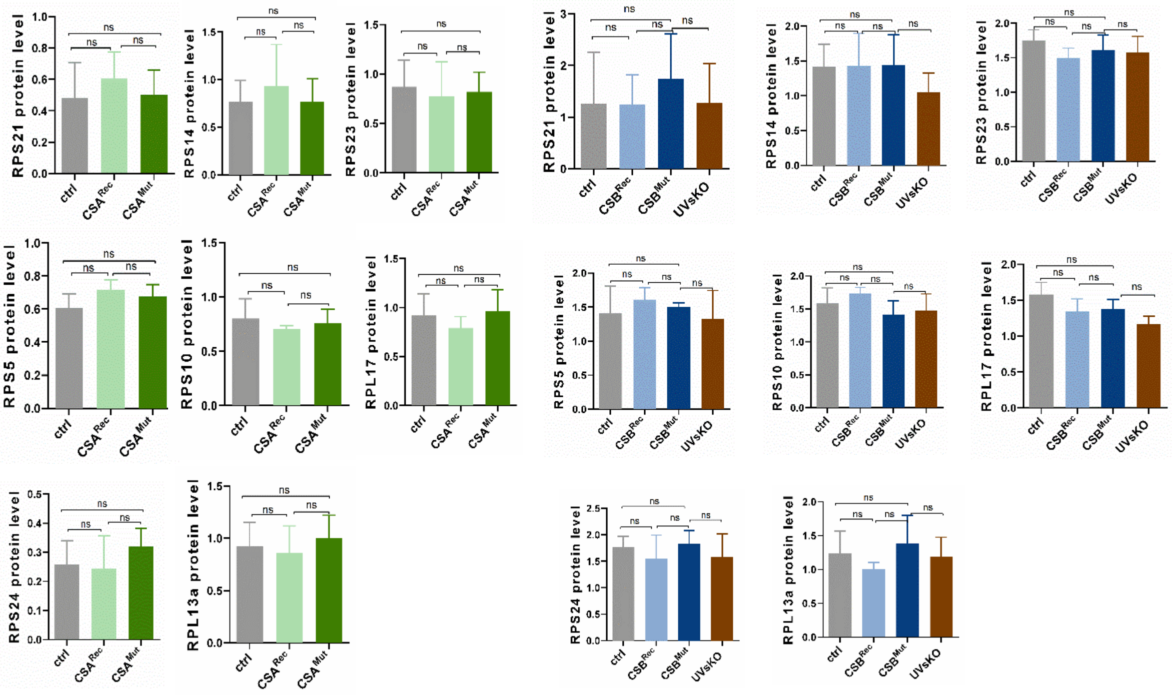

B

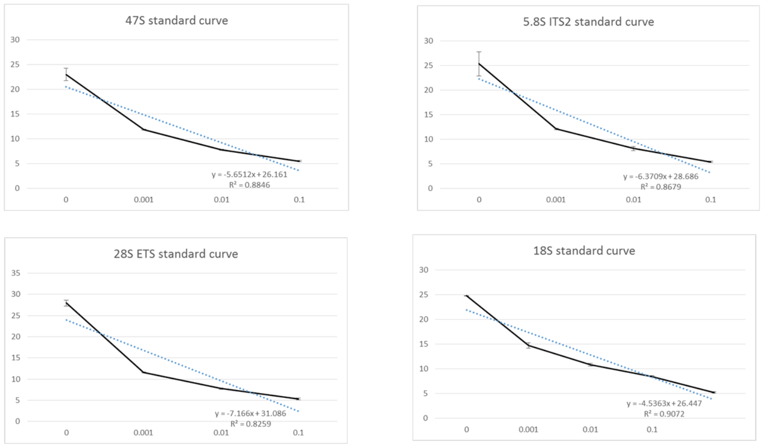

Figure S3 A. Quantification of Western Blots of ribosomal proteins in lysates. Values are represented as mean ± SD of at least three independent experiments (*=p<0.05, **=p<0.01, ***=p<0.001) **B.** Standard curves for qPCR
